# Performance of a Novel Blood-Based Early Colorectal Cancer Screening Assay in Remaining Serum after the Blood Biochemical Test

**DOI:** 10.1155/2019/5232780

**Published:** 2019-04-04

**Authors:** Ying Chen, Zhenzhen Wang, Guodong Zhao, Chuang Sun, Yong Ma, Linyan Zhang, Minxue Zheng, Hongchun Li

**Affiliations:** ^1^School of Medical Technology, Xuzhou Medical University, Xuzhou Jiangsu 221004, China; ^2^Suzhou Institute of Biomedical Engineering and Technology, Chinese Academy of Sciences, Suzhou Jiangsu 215163, China; ^3^Zhejiang University Kunshan Biotechnology Laboratory, Zhejiang University Kunshan Innovation Institute, Kunshan Jiangsu 215300, China; ^4^Suzhou VersaBio Technologies Co. Ltd., Kunshan Jiangsu 215300, China; ^5^Department of Laboratory Medicine, Affiliated Hospital of Xuzhou Medical University, Xuzhou 221002, China

## Abstract

**Background:**

Combination of multiple biomarkers was an effective strategy to improve sensitivity in cancer diagnosis and screening. However, the performance of the combination of methylated *SEPT9* and *SDC2* for detection of colorectal cancer (CRC) has yet to be reported.

**Methods:**

A new qPCR-based assay combining the detection of methylated *SEPT9* and *SDC2* was used. Methylation statuses of *SEPT9* and *SDC2* were examined in 19 sets of cancer tissues and paired adjacent tissues and further evaluated with 225 serum samples, including 111 CRC patients and 114 no evidence of disease individuals.

**Results:**

*SEPT9* and *SDC2* methylation levels were higher in 94.7% and 100.0% of cancer tissues than in their paired adjacent tissues. The sensitivities for detecting CRC by *SEPT9* methylation alone and *SDC2* methylation alone were 73.0% (95% CI: 63.6–80.8%) and 71.2% (95% CI: 61.8–79.2%), respectively, with the same specificity of 95.6% (95% CI: 89.6–98.4%). However, when *SEPT9* methylation was combined with *SDC2* methylation to detect CRC, the sensitivity was improved to 86.5% (95% CI: 78.4–92.0%) with a specificity of 92.1% (95% CI: 85.1–96.1%).

**Conclusion:**

The combination of methylated *SEPT9* and *SDC2* detection in serum has the potential to be a noninvasive strategy for CRC screening.

## 1. Introduction

Colorectal cancer (CRC) is among the three most common cancer types worldwide [1]. It is also one of the five most common cancer types in China [2]. And due to the changes towards a more westernized lifestyle of Chinese population, its incidence has seen steady increase in recent years. During the past decade, the 5-year relative survival rate of Chinese CRC patients has increased from 47.2% to 56.9%, which, however, was still more than 8% lower than that of the developed countries [3, 4].

The Chinese CRC screening guideline recommends screening adults of 40–74 years old with the guaiac fecal occult blood test (gFOBT) followed up with digital rectal exam and colonoscopy [1]. However, it has been very difficult for the program to reach the entire population, resulting in a very low screening uptake. Due to invasiveness and bothersome bowel preparation, colonoscopy has rarely been used despite being the gold standard for CRC screening [5]. Moreover, it is hardly a primary CRC screening method in developing countries with limited resources including China where gFOBT is the most widely used CRC screening method albeit with low accuracy [6]. Hence, a noninvasive and more accurate screening method needs to be developed to promote early CRC screening.

DNA methylation, the addition of methyl groups to the cytosine residues of DNA, is a form of epigenetic modification that mainly occurs in CpG islands usually present in promoter regions [7]. Abnormal hypermethylation of certain CpG islands may lead to transcriptional silencing and inactivation of cancer suppressor genes [8]. In recent years, several studies have reported the application of DNA methylation biomarkers in CRC diagnosis and screening [5, 9]. *SEPT9* methylation, the only blood-based biomarker approved by FDA for CRC screening, has been used clinically for nearly 10 years [10, 11]. It has been proven an accurate, reliable, fast, and convenient method for CRC early screening [12]. However, the sensitivity of *SEPT9* methylation for CRC detection was relatively low, especially for early stage cancers [3, 4]. *Syndecan-2* (*SDC2*) gene was also found to be hypermethylated in the feces or blood samples of most CRC patients [10, 13, 14].

Biochemical tests are the most common medical testing program in clinics; they measure sodium, potassium, chloride, bicarbonate, blood urea nitrogen, and so on. For the majority of biochemical tests, blood is usually obtained from the patient's vein, and serum or plasma is obtained from the blood sample for the next step test. However, most of the serum or plasma is discarded after the biochemical test is completed. Therefore, use of the remaining serum or plasma after the blood biochemical test might be an effective approach to detect CRC.

Combination of multiple biomarkers and/or methods has become a trend in cancer diagnosis and screening to improve sensitivity [15]. For example, it was reported that the sensitivities for CRC detection were 72.2% and 68.0%, respectively, for *SEPT9* methylation and the fecal immunochemical test (FIT) individually, and the specificities were 81.5% and 97.4%. When test results for *SEPT9* methylation and FIT were combined, the CRC detection rate was 88.7% with a specificity of 78.8% [16]. However, the combination of *SEPT9* and *SDC2* methylation for CRC screening has never been reported. In this study, we evaluated the performance of a new blood-based early CRC screening assay (ColoDefense), which combined the detection of *SEPT9* and *SDC2* methylations in a single qPCR reaction, with the remaining serum samples after the blood biochemical test. Such an approach also has an added advantage of avoidance of repeated blood draw.

## 2. Materials and Methods

### 2.1. Sample Collection

Fresh-frozen cancer tissues (*n* = 19) and paired adjacent paracancer tissues (*n* = 19) from CRC patients were collected at the time of surgery at the Affiliated Hospital of Xuzhou Medical University. Serum specimens were collected from 111 CRC patients undergoing colonoscopy at the same hospital and histologically confirmed by a pathologist. Control serum specimens were collected from 114 subjects with no evidence of diseases (NED). The remaining serum specimens after the blood biochemical test were collected and frozen at -80°C until used. The study was approved by the Institutional Review Board of the Affiliated Hospital of Xuzhou Medical University (Ethics Committee reference number: XYFY2017-KL105-02), and the informed consent was obtained from all participating patients and healthy control subjects.

### 2.2. DNA Extraction, Bisulfite Treatment, and Quantitative Real-Time PCR

Genomic DNA was isolated from tissue specimens using a DNA mini kit (Qiagen). For serum samples, the volume was adjusted to 3.5 mL with PBS buffer if the serum volume was less than 3.5 mL, and each sample was extracted using a cfDNA extraction kit (Suzhou VersaBio Technologies Co. Ltd.). Subsequently, bisulfite conversion of purified DNA and purification of the converted product were performed with a bisulfite conversion kit (Suzhou VersaBio Technologies Co. Ltd.). All the kits were used according to the manufacturers' instructions.

Purified DNA obtained from the above steps was tested by real-time methylation-specific qPCR using the ColoDefense test (Suzhou VersaBio Technologies Co. Ltd.), a new blood-based methylation assay for colorectal cancer screening. For the ColoDefense assay, methylated *SEPT9*, methylated *SDC2*, and an internal control (*ACTB*) were performed in the same reaction. Three separate PCR replicates were prepared with purified bisulfite-converted DNA from each serum sample, and single PCR reaction was performed with purified bisulfite-converted DNA from each tissue sample. Real-time PCR was performed on the LC480-II thermal cycler (Roche Diagnostics) using the following cycling conditions: activation at 95°C for 30 minutes, 50 cycles at 95°C for 10 seconds and 56°C for 30 seconds, and final cooling at 40°C for 30 seconds.

### 2.3. Data Analysis

∆Cp was used to determine the tissue methylation status of *SDC2* and *SEPT9*; ∆Cp was defined as the difference between the Cp values for the target (methylated *SDC2* or methylated *SEPT9*) and the internal control gene (*ACTB*). The results for serum specimens were “invalid” if the *ACTB* Cp was greater than 35.0, and methylated *SEPT9* and methylated *SDC2* were “detected” if their Cp values were less than 50.0. Methylated *SEPT9* was analyzed by using a 1/3 rule in which a serum sample was scored positive if one of the three PCR replicates had a valid amplification curve (1/3 algorithm). And methylated *SDC2* was analyzed by using a 2/3 rule, whereby to be called positive, two of three PCR replicates of a serum sample must have valid amplification curves (2/3 algorithm). The serum sample would be considered as positive if either methylated *SEPT9* or methylated *SDC2* was positive. Data were subjected to statistical analysis by using IBM SPSS for Windows version 22.0, and the *t* test was used for comparison between two samples at the significant level of *p* < 0.01. Data from sensitivity and specificity were used to plot the receiver operating characteristic (ROC) curve. Because most Cp values from normal individuals were not detected in the qPCR reaction, we had to set the Cp values to 50.0 (the maximal number of PCR cycles) for those not detected samples to plot the curve [11].

## 3. Results

**Figure 1:**
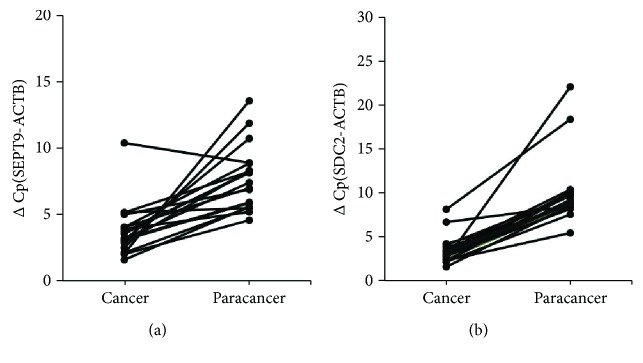
*SEPT9* and *SDC2* methylation levels in colorectal cancer tissues and paracancer tissues. ∆Cp was defined as the difference between the Cp values for the target (methylated *SEPT9* or methylated *SDC2*) and the internal control gene (*ACTB*): (a) methylation levels of *SEPT9* and (b) methylation levels of *SDC2*.

The ColoDefense assay was used to quantify methylation levels of *SEPT9* and *SDC2* genes in 19 colorectal cancer tissues and paired adjacent paracancer tissues. The *SEPT9* and *SDC2* methylation levels were higher in 94.7% (18/19) and 100.0% (19/19) of cancer tissues than in their paired adjacent paracancer tissues (*p* < 0.001, Figure 1), thus making the ColoDefense assay a candidate method for CRC screening.

To examine the feasibility of applying the ColoDefense assay in CRC screening, 225 remaining serum samples after the blood biochemical test were collected from the Affiliated Hospital of Xuzhou Medical University, of which 111 were from CRC patients. The ages of all CRC patients ranged from 25 to 89 with a mean age of 61 and a median age of 62. The ages of NED individuals ranged from 19 to 60. The volumes of all serum samples ranged from 0.5 to 3.5 mL. The median serum volumes for both CRC patients and NED individuals were 1.8 mL (Table 1).

**Figure 2:**
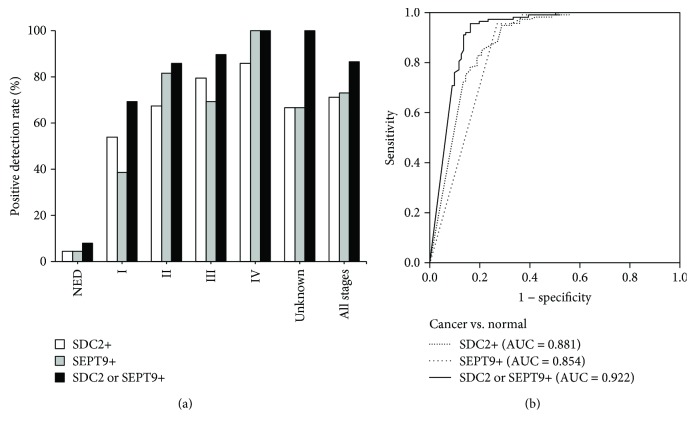
Sensitivity of ColoDefense in detecting colorectal cancer across stages I-IV with serum samples: (a) positive detection rates for NED individuals and all stages of colorectal cancer and (b) ROC curves for the ColoDefense in detecting colorectal cancer.

When the serum samples were subjected to the ColoDefense assay, the positive detection rate for each stage of CRC is shown in Figure 2(a). The sensitivity for detecting CRC by methylated *SEPT9* alone was 73.0% (95% CI: 63.6–80.8%) with a specificity of 95.6% (95% CI: 89.6–98.4%). The sensitivity by methylated *SDC2* alone was 71.2% (95% CI: 61.8–79.2%) with the same specificity of 95.6% (95% CI: 89.6–98.4%). However, when methylated *SEPT9* was combined with methylated *SDC2* to detect the CRC, the overall sensitivity was improved to 86.5% (95% CI: 78.4–92.0%) with a specificity of 92.1% (95% CI: 85.1–96.1%).

In the 108 cases of CRC whose stages were identified based on the surgically resected specimens, methylated *SEPT9* alone was positive in 38.5% of stage I (5/13), 81.6% of stage II (40/49), 69.2% of stage III (27/39), and 100% of stage IV (7/7). Methylated *SDC2* alone was positive in 53.9% of stage I (7/13), 67.4% of stage II (33/49), 79.5% of stage III (31/39), and 85.7% of stage IV (6/7). However, for the methylated *SEPT9* and methylated *SDC2* panel (ColoDefense), the sensitivities for different stages of CRC were 69.2% for stage I (9/13), 85.7% for stage II (42/49), 89.7% for stage III (35/39), and 100% for stage IV (7/7) (Figure 2(a)).

The ROC curves for ColoDefense detecting CRC in serum are shown in Figure 2(b). The AUC for methylated *SEPT9* alone was 0.854 (95% CI: 0.800–0.907), and the AUC for methylated *SDC2* alone was 0.881 (95% CI: 0.835–0.928). However, the methylated *SEPT9* and methylated *SDC2* panel (ColoDefense) improved the AUC to 0.922 (95% CI: 0.883–0.961), indicating both high sensitivity and high specificity of the assay and a good performance in distinguishing the CRC subjects from NED subjects.

Due to the inconsistent volume of serum samples in this study, serum samples were divided into three groups by volume (<1.5 mL, 1.5-2.5 mL, and >2.5 mL) for further analysis (Table 2). There was no increase in the positive detection rate as the volume increased for different stages when detected by methylated *SEPT9* alone, methylated *SDC2* alone, or the methylated *SEPT9* and methylated *SDC2* panel (ColoDefense), and there seemed to be no significant difference among the positive detection rates of different volume groups (*p* > 0.01, data not shown).

Furthermore, there was no significant difference among the positive detection rates of methylated *SEPT9* alone or methylated *SDC2* alone or a combination of methylated *SEPT9* and methylated *SDC2* between different ages, genders, or locations of the tumors (*p* > 0.01, Table 3). However, the positive detection rates seemed to increase with the increase of tumor sizes. The positive detection rates were 36.4%, 72.4%, and 94.7%, respectively, for tumors of <3 cm, 3-6 cm, and >6 cm in sizes by methylated *SEPT9* alone, 54.6%, 69.7%, and 89.5% by methylated *SDC2* alone, and 63.6%, 85.5%, and 100.0% by the methylated *SEPT9* and methylated *SDC2* panel (ColoDefense).

## 4. Discussion

CRC is a leading cause of cancer-related deaths worldwide, and late-stage CRC patients have low five-year survival rates. Blood-based screening strategies present the advantage of minimal invasiveness compared to endoscopies and are expected to have higher compliance rates than stool-based tests [10]. At present, a blood-based *SEPT9* methylation assay for CRC screening, the Epi proColon 2.0 assay, has been approved by FDA and Chinese FDA (CFDA) [17]. Epi proColon 2.0 showed a sensitivity of 68.2% and a specificity of 78.2% in a large cohort study (1544 plasma samples) with the 1/3 algorithm [18]. Another clinical trial reported that the sensitivities of Epi proColon 2.0 for detecting stages I–IV CRC were 35.0%, 63.0%, 46.0%, and 77.4%, respectively [19]. And several case-control studies reported that the sensitivity of Epi proColon 2.0 for detecting CRC ranged from 73.3% to 81.0% [16, 17]. Overall, although methylated *SEPT9* was the only blood-based biomarker approved by FDA for CRC screening, its sensitivity did not show obvious advantage over FIT [20]. The combination of methylated *SEPT9* with FIT or CEA could significantly enhance the sensitivity of CRC detection compared with any assay alone [19]. However, the specificity was significantly decreased due to the accumulation of false-positive cases from each biomarker. Therefore, the best combination should be the one that best balances sensitivity and specificity.

Methylated *SDC2* as a new blood-based biomarker for CRC was noticed in recent years. Two recent studies reported that the sensitivity of methylated *SDC2* for CRC screening with serum or plasma samples was 87.0% with a specificity of 95.2% [13] or 89.4% with a specificity of 81.1% [9], respectively. The sensitivities of methylated *SDC2* from these studies were improved by nested PCR or calculation of the percentage of methylated *SDC2* with *ACTB* as the reference gene. In this study, we introduced a new blood-based early CRC screening assay, ColoDefense, which combined two methylation biomarkers, *SEPT9* and *SDC2*, in a single PCR reaction. Compared with Epi proColon 2.0, methylated *SEPT9* alone from ColoDefense showed similar sensitivity and specificity, and both had relatively low positive detection rates for stages I and III CRC (35.0% [4] vs. 38.5% and 46.0% [4] vs. 69.2%). On the other hand, methylated *SDC2* alone from ColoDefense showed higher positive detection rates for stages I and III CRC (53.9% and 79.5%). Taken together, the combination of methylated *SEPT9* and methylated *SDC2* significantly improved the positive detection rates for stages I and III CRC (Figure 2(a)). The overall sensitivity of methylated *SEPT9* alone or methylated *SDC2* alone was 73.0% or 71.2%, respectively, with specificity of 95.6% for both. In contrast, the combination of methylated *SEPT9* and methylated *SDC2* increased sensitivity to 86.5% with a specificity of 92.1%, resulting in 13.5% and 15.3% increases in sensitivity compared to methylated *SEPT9* alone and methylated *SDC2* alone whereas specificity only decreased 3.5% (Figure 2).

For Epi proColon 2.0, real-time PCR reactions are performed with 3.5 mL fresh plasma samples as initial input, which means that approximately 10 mL of blood is needed for each test. As the blood biochemical test is the most common test in the clinics and large amount of serum remains after each test, using the remaining serum samples for CRC screening may avoid repeated blood draw. In this study, we found that the ColoDefense assay on such serum samples did show high sensitivity and specificity for CRC detection. In particular, by classifying serum samples by volume, it was found that serum volume was not the most important factor affecting CRC positive detection rates (Table 2). Therefore, using less blood volume, such as 5 mL, or remaining serum samples after the blood biochemical test might be a promising CRC screening strategy for those patients who refuse repeated blood draw or are unwilling to have 10 mL blood drawn.

## 5. Conclusion

In this study, we evaluated a new blood-based CRC early screening assay, ColoDefense, which combines two methylation biomarkers, *SEPT9* and *SDC2*. The results demonstrated that the CRC positive detection rates were significantly improved by the methylated *SEPT9* and methylated *SDC2* panel without significant impact on specificity. It suggested that the methylated *SEPT9* and methylated *SDC2* panel might be the best combination for early CRC screening with high sensitivity and specificity.

## Figures and Tables

**Table 1:** Characteristics of individuals examined by the ColoDefense assay.

	CRC	NED
*Age (years)*		
Min–Max	25-89	19-60
Mean ± SD	61.0 ± 12.0	33.2 ± 8.3
Median	62	32
*Serum volume (mL)*		
Min–Max	0.5-3.5	0.7-3.5
Mean ± SD	2.16 ± 1.01	1.45 ± 0.85
Median	1.8	1.8

CRC: colorectal cancer; NED: no evidence of diseases.

**Table 2:** Results of ColoDefense in detecting colorectal cancer between different serum volumes.

Stages	Serum volume (mL)	*n*	*SDC2*+ (%)	*SEPT9*+ (%)	*SDC2* or *SEPT9*+ (%)
I	<1.5	3	66.7 (2/3)	33.3 (1/3)	100.0 (3/3)
	1.5-2.5	4	25.0 (1/4)	25.0 (1/4)	50.0 (2/4)
	>2.5	6	66.7 (4/6)	50.0 (3/6)	66.7 (4/6)

II	<1.5	16	62.5 (10/16)	75.0 (12/16)	81.3 (13/16)
	1.5-2.5	11	36.4 (4/11)	81.8 (9/11)	81.8 (9/11)
	>2.5	22	86.4 (19/22)	86.4 (19/22)	90.9 (20/22)

III	<1.5	10	90.0 (9/10)	70.0 (7/10)	100.0 (10/10)
	1.5-2.5	11	63.6 (7/11)	63.6 (7/11)	72.7 (8/11)
	>2.5	18	83.3 (15/18)	72.2 (13/18)	94.4 (17/18)

IV	<1.5	4	75.0 (3/4)	100.0 (4/4)	100.0 (4/4)
	1.5-2.5	3	100.0 (3/3)	100.0 (3/3)	100.0 (3/3)
	>2.5	NA	NA	NA	NA

Unknown	<1.5	2	50.0 (1/2)	100.0 (2/2)	100.0 (2/2)
	>2.5	1	100.0 (1/1)	NA	100.0 (1/1)

Total stages	<1.5	35	71.4 (25/35)	74.3 (26/35)	91.4 (32/35)
	1.5-2.5	29	51.7 (15/29)	69.0 (20/29)	75.9 (22/29)
	>2.5	47	83.0 (39/47)	74.5 (35/47)	89.4 (42/47)

NA: not applicable.

**Table 3:** Results of ColoDefense in detecting colorectal cancer between different ages, genders, tumor locations, and tumor sizes.

	*SDC2*+ (%)	*p* value	*SEPT9*+ (%)	*p* value	*SDC2* or *SEPT9*+ (%)	*p* value
*Age*						
<60 (*n* = 38)	86.8 (33/38)	0.01	63.2 (24/38)	0.09	89.5 (34/38)	0.51
≥60 (*n* = 73)	63.0 (46/73)		78.1 (57/73)		84.9 (62/73)	
*Gender*						
Male (*n* = 75)	73.3 (55/75)	0.47	74.7 (56/75)	0.56	88.0 (66/75)	0.50
Female (*n* = 36)	66.7 (24/36)		69.4 (25/36)		83.3 (30/36)	
*Location*						
Rectum (*n* = 60)	68.3 (41/60)	0.45	66.7 (40/60)	0.15	86.7 (52/60)	0.85
Cecum (*n* = 48)	75.0 (36/48)		79.2 (38/48)		85.4 (41/48)	
NA (*n* = 3)	66.7 (2/3)		100.0 (3/3)		100.0 (3/3)	
*Size*						
<3 cm (*n* = 11)	54.6 (6/11)		36.4 (4/11)		63.6 (7/11)	
3-6 cm (*n* = 76)	69.7 (53/76)		72.4 (55/76)		85.5 (65/76)	
>6 cm (*n* = 19)	89.5 (17/19)		94.7 (18/19)		100.0 (19/19)	
NA (*n* = 5)	100.0 (5/5)		80.0 (4/5)		100.0 (5/5)	

NA: not applicable.

## Data Availability

The data used to support the findings of this study are included within the article.
